# Dysfunctional mitochondrial respiration in the striatum of the Huntington’s disease transgenic R6/2 mouse model

**DOI:** 10.1371/currents.hd.d8917b4862929772c5a2f2a34ef1c201

**Published:** 2013-04-02

**Authors:** Frederik Heurlin Aidt, Signe Marie Borch Nielsen, Jørgen Kanters, Dominik Pesta, Troels Tolstrup Nielsen, Anne Nørremølle, Lis Hasholt, Michael Christiansen, Christian Munch Hagen

**Affiliations:** Institute of Cellular and Molecular Medicine, Faculty of Health Sciences, University of Copenhagen; Section of Molecular Medicine, Department of Clinical Biochemistry, Genetics and Immunology, Statens Serum Institut, Copenhagen, Denmark; Institute of Cellular and Molecular Medicine, Faculty of Health Sciences, University of Copenhagen; Department of Biomedical Sciences, Faculty of Health Sciences, University of Copenhagen, Copenhagen, Denmark; Department of Radiology, Innsbruck Medical University, Innsbruck, Austria; Danish Dementia Research Center, Department of Neurology, Rigshospitalet, Copenhagen University Hospital, Denmark; Institute of Cellular and Molecular Medicine, Faculty of Health Sciences, University of Copenhagen; Institute of Cellular and Molecular Medicine, Faculty of Health Sciences, University of Copenhagen; Section of Molecular Medicine, Department of Clinical Biochemistry, Genetics and Immunology, Statens Serum Institut, Copenhagen, Denmark

## Abstract

Metabolic dysfunction and mitochondrial involvement are recognised as part of the pathology in Huntington's Disease (HD). Post-mortem examinations of the striatum from end-stage HD patients have shown a decrease in the in vitro activity of complexes II, III and IV of the electron transport system (ETS). In different models of HD, evidence of enzyme defects have been reported in complex II and complex IV using enzyme assays. However, such assays are highly variable and results have been inconsistent. We investigated the integrated ETS function ex vivo using a sensitive high-resolution respirometric (HRR) method. The O2 flux in a whole-cell sample combined with the addition of mitochondrial substrates, uncouplers and inhibitors enabled us to accurately quantitate the function of individual mitochondrial complexes in intact mitochondria, while retaining mitochondrial regulation and compensatory mechanisms. We used HRR to examine the mitochondrial function in striata from 12-week old R6/2 mice expressing exon 1 of human HTT with 130 CAG repeats. A significant reduction in complex II and complex IV flux control ratios was found in the R6/2 mouse striatum at 12 weeks of age compared to controls, confirming previous findings obtained with spectrophotometric enzyme assays.

## Introduction

HD is a rare hereditary fatal neurodegenerative disease, with a prevalence of 5-6 per 100.000 in Europe and North America. It is caused by an expanded stretch of >36+ CAG repeats in exon 1 of the *HTT* gene 1. The expansion length inversely correlates with age of onset 2. Wild type huntingtin protein (Htt) is ubiquitously expressed, although the expression is especially high in the testis and brain, in particular in the striatum, cortex and hippocampus 3. The major neuropathological phenotype of HD is a loss of a specific striatal neuronal subpopulation, medium spiny neurons, resulting in striatal atrophy 4. However, the disease also manifests systemically. Metabolic dysfunction has long been recognised as part of the pathology in HD, underlined by the weight loss of HD patients 5. Decreased glucose metabolism and increased lactate concentrations in several brain regions of symptomatic HD patients 6 ^,^ 7 ^,^ 8 ^,^ 9 indicate mitochondrial involvement in the disease. Htt destabilises the mitochondrial outer membrane, which increases the sensitivity of the mitochondrial transition pore to Ca^2+^ and other apoptotic stimuli 10 ^,^ 11. Moreover, mitochondria in HD models have been shown to be dysfunctional with respect to fission and fusion 12 ^,^ 13 , trafficking 14 ^,^ 15 , cell respiration and ATP production 16.

Impairment of the electron transport system (ETS) in HD is an area of controversy in HD research 17 ^,^ 18. The ETS consists of the respiratory complexes I, II, III and IV (CI, CII, CIII and CIV), which generate the proton motive force. This is utilised mainly to generate ATP and mitochondrial Ca^2+^ uptake 19. Post-mortem examinations of the striatum have demonstrated a decrease in the *in vitro* enzyme activity of CII, CIII and CIV only in the late stage disease 20 ^,^ 21 ^,^ 22.

With the emergence of transgenic HD models, enzyme activity could be examined without the risk of post-mortem brain tissue modifications. However, conflicting results have been obtained from such studies. Whilst several studies have described dysfunction in CII and CIV, others report intact function (summarised in Table 1). In addition, several reviews mention unpublished data that purportedly could not reproduce any ETS deficiency in a number of mouse models 18 ^,^ 23 ^,^ 24 ^,^ 25. One of the findings disputed in these reviews is the study by Tabrizi et al. where CIV deficiency was reported in the R6/2 transgenic mouse striatum 26. The R6/2 mouse expresses an N-terminal fragment of mutant human *HTT* exon 1 with ~130 CAG repeats. The phenotype is characterised by the emergence of HD symptoms at ~9 weeks of age, with a severe phenotype at 12 weeks of age and premature death at around 13 weeks of age 27 . In the previous studies of the ETS function in R6/2 mice, the activities of the individual complexes were evaluated using enzyme assays. These methods have since been demonstrated to confer a considerable methodological variation 28 ^,^ 29. In addition, the ETS is organised in supercomplexes (respirasomes) and measurement of single enzyme activities does not provide an accurate description of the integrated ETS function 30.

**Table 1. Overview of reported ETS dysfunction in HD models.** 

**Model name**	**Transgenic insert**	**Complexes affected**	**Method**	**Reference**
HD89 and HD48 mouse	human *HTT* gene coding for full-length Htt with 89 CAG repeats	None	Spectrophotometric enzyme assay.	20
R6/2 mouse (12 weeks)	N-terminal fragment model with 115 CAG repeats	CIV, aconitase	Spectrophotometric enzyme assay.	26
R6/2 mouse (8 weeks)	N-terminal fragment model with 115 CAG repeats	None	Autoradiogram.	25
N171-82Q mouse (20 weeks)	N-terminal fragment model with 82 CAG repeats	CIV	Spectrophotometric enzyme assay	37
Wistar rats (8 weeks post-injection)	Lentiviral N-terminal fragment model with 82 CAG repeats	CII	Autoradiogram	38
Wistar rats	N-terminal fragment with 52 CAG repeats	CI+II_OXPHOS _flux	High-resolution respirometry	39
HdhQ111 striatal cells	Knock-in mouse model, carries 111 CAG repeats in endogenous *HTT* gene	None	Respirometry	47
Neonatal striatal HdhQ^150^ cells	Knock-in mouse model, carries 150 CAG repeats in endogenous *HTT* gene	None	Respirometry	17
Hela cells	Transient expression of N-terminal *HTT* fragment with 40 CAG repeats	CII	Spectrophotometric enzyme assay	40
Rat embryonic striatal neurons	Lentivirally transduced with N-terminal fragment with 82 CAG repeats	CII subunit concentration reduced	Western blot	41
R6/2 mouse, N171-82Q YAC-72 mouse, *Hdh* ^Q92 ^mouse, *Hdh* ^Q111^ mouse	-	None	N/A	22 ^,^ 23 ^,^ 24

In order to investigate the reported ETS dysfunction in the R6/2 mouse model using more sensitive methods, we analysed the mitochondrial function in the striatum of 12-week old R6/2 female transgenic mice and wild type littermates using HRR. In contrast to spectrophotometric enzyme assays, HRR takes into account the entire spectrum of respiratory control, compensatory mechanisms and cellular architecture affecting mitochondrial function as the respirometric measurements are carried out on non-isolated mitochondria that that retain their proper cellular context. Whilst isolation of mitochondria has been shown to drastically influence function 31, tissue homogenate HRR has been demonstrated to constitute a sensitive method for analysing the integrated mitochondrial function 32.

## Materials and methods

Animal studies

R6/2 mice, transgenic for exon 1 gene of the human HD gene containing approximately 130 CAG repeat units 27, originated from the Jackson Laboratory (Bar Harbor, Maine) and were maintained by backcrossing males to CBA/J x B6 females (Taconic, Denmark). The behavioural phenotype of the colony has been described previously 33. The mice were kept under specific pathogen free (SPF) conditions at a 12-hour light / 12-hour darkness cycle in standard polystyrene cages with free access to standard chow. Tail tip DNA was used for genotyping 34. The CAG repeat lengths of mice from the colony were around 130 throughout the experiment. In each experiment, we used six 12-week-old female R6/2 mice and six control female littermates without the HD transgene. The experiment was performed twice, yielding a total of 12 animals in each group. Experiments were performed in accordance with and approved by the Danish Animal Experiments Inspectorate.

Sample preparation

Experimental animals were sacrificed by cervical dislocation. The brains were excised and placed in ice-cold mitochondrial respiration medium MiR05 (EGTA 0,5 mM, MgCl_2_ 3 mM, K-lactobionate 60 mM, taurine 20 mM, KH_2_PO_4_ 10 mM, HEPES 20 mM, sucrose 110 mM, BSA 1 g/L, adjusted to pH 7.1) 35. The striatum was then dissected, weighed and homogenised in a pre-cooled mortar with a pestle in MiR05 medium. The crude homogenate was filtered through a 40 µm cell strainer (BD Falcon, San Jose, CA, USA). All chemicals were purchased from Sigma-Aldrich (St. Louis, MO, USA).

High-resolution respirometry

Mitochondrial respiration was measured in a high-resolution oxygraph (Oxygraph-2k, Oroboros Instruments, Innsbruck, Austria) at 37°C. Striatum homogenates (5-10 mg) were suspended in 2 mL MiR05 medium. Oxygen concentration (µM = nmol/ml) and oxygen ﬂux (pmol/(s·ml) was recorded online using DatLab software version 4.3.2.7 (Oroboros Instruments, Innsbruck, Austria).

Experimental protocol

The striatum homogenate was suspended in MiR05, added to the Oxygraph-2k glass chambers and the O_2_ flux was allowed to stabilise. A substrate, uncoupler, inhibitor titration (SUIT) protocol was applied to assess qualitative and quantitative mitochondrial changes in R6/2 transgenic mice and unaffected controls. After stabilisation, LEAK respiration was evaluated by adding the CI substrates malate (0.8 mM), pyruvate (2 mM) and glutamate (10 mM). The maximum oxidative phosphorylation (OXPHOS) capacity with CI substrates was attained by the addition of ADP+Mg^2+^ (2.25 mM) (CI_OXPHOS_). For quality control of mitochondrial integrity cytochrome c (CytC) (10 mM) was added (CI_OXPHOS_+CytC). For evaluation of maximum OXPHOS capacity of the convergent input from CI and CII at saturating ADP-concentration, the CII substrate succinate (10 mM) was added (CI+CII_OXPHOS_). Maximum ETS capacity was obtained by stepwise titration of the uncoupler carbonylcyanide p-trifluoromethoxyphenyl-hydrazon (FCCP, 1 pmol/step) (CI+II_ETS_). Rotenone (2.5µM) was added to inhibit CI; hence the maximal ETS capacity supported by CII alone was determined (CII_ETS_). Residual oxygen consumption (ROX) was established by addition of the CIII inhibitor Antimycin A (2.5 mM). Finally, maximal CIV activity was determined by addition of 0.5 mM TMPD (N,N,N,N-Tetramethyl-p-phenylenediaminedihydrochloride), a substrate for the reduction of CytC, and 2 mM ascorbate (CIV_max_). CIV_max _was corrected for autoxidation of substrates as previously described 36. ROX was subtracted from the fluxes in each run to correct for non-mitochondrial respiration. All samples were run in duplicates and the mean was used for analysis. The mean variation between duplicates was 0.9% ± 0.7% SEM for wild type and 2.3% ± 1.2% SEM for R6/2 measurements.

Citrate synthase assay

Protein was extracted from 2.5 mg striatal homogenate using CelLyticM (Sigma-Aldrich, St. Louis, MO, USA) containing 1X Complete protease inhibitor cocktail (Roche, Basel, Switzerland). Citrate synthase (CS) assay was performed in duplicate using the Citrate Synthase Assay Kit (Sigma-Aldrich, St. Louis, MO, USA) and the mean was used for analysis. The mean variation between duplicates was 3.6% ± 3.48% SEM. Samples were analysed in a Victor3 1420 multiplate reader (Perkin Elmer, Waltham, MA, USA) at l_abs_= 412 nm.

Data analysis

D'Agostino & Pearson omnibus normality test was applied to all data sets. Student´s *t*-test was used for statistical analysis. The significance level was set at p<0.05. Differences were considered trends if p<0.1. Graphs were generated with GraphPad Prism 5 (GraphPad Software, Inc., La Jolla, CA, USA). All differences are given as the mean difference ± the standard error of the mean difference.

## Results

The integrated ETS pathway was assessed using HRR by determining the flux control ratios (FCR) in the striatum of R6/2 transgenic mice and wild type littermates using a SUIT protocol. The FCRs were generated by normalising each respiratory state to the maximal uncoupled mitochondrial respiration, CI+II_ETS _(State 3_U_). The respiratory control ratio (RCR) was generated by normalising CI+CII_OXPHOS_ to LEAK (State 3_ADP_/state 4). The main findings were significant quantitative differences in CI+II_OXPHOS_, CII_ETS_ and CIV_max_ when expressed as FCRs (Figure 1). The mean CI+II_OXPHOS_ FCR was decreased in the R6/2 mice relative to wild type controls by 0.035 ± 0.011 (5.2% ± 1.6%), whilst the mean CII_ETS _FCR was decreased by 0.041 ± 0.018 (11.7% ± 5.1%). The mean CIV_max_ value was decreased by 0.149 ± 0.07 (11.1% ± 5.2%). No significant differences in CI_OXPHOS_(0.9% ± 4.6%) or in LEAK (2.1% ± 9.3%) were seen. The mean SD of the generated FCRs was 10.5%.

**High-resolution respirometry data showing flux control ratios of the respiratory states normalised to the electron transport system (ETS) capacity (CI+CIIETS).**
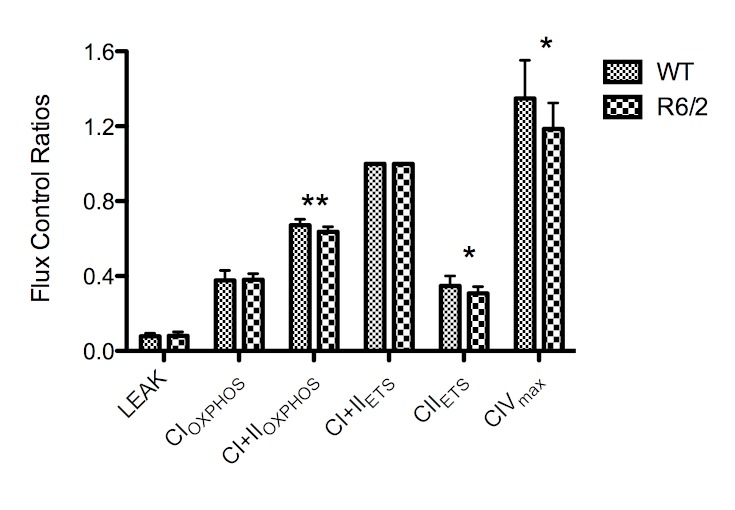
n=12 for each group. Error bars depict SD. Significance is denoted by p<0.05* and p<0.01**.

No significant increase of flux was observed after addition of cytochrome *c* (WT FCR difference = -1.3% ± 5.8%, R6/2 FCR difference = 0.56% ± 3.5%), indicating an adequate quality of mitochondrial preparations. The RCR was not significantly decreased in the R6/2 mice compared to wild type (-5.2% ± 9.1%) (Figure 2).

**Respiratory control ratios (RCR) (ratio between CI+CIIOXPHOS and LEAK or state 3/state4).**
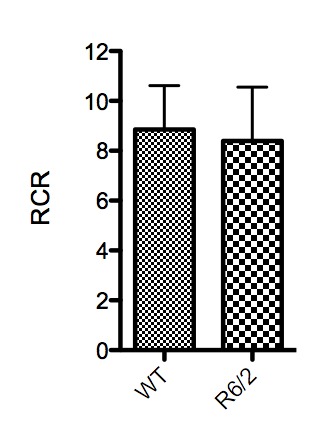
n=12 for each group. No significant difference between groups was found. Error bars depict SD.

We measured the CS activity/mg tissue in the samples. We found that there was no significant difference in CS activity (p=0.74) or variation (p=0.45) in wild-type mice compared to R6/2 transgene mice. As populations were not significantly different and variances were equal, we pooled all CS activities to get an estimate of the analytical variation, and calculated the SD as ± 38,5%.

Normalising the O_2_ flux to CS activity failed to demonstrate any significant differences in O_2_ flux per unit CS, although a trend (p<0.1) similar to the observed decreases in R6/2 mice FCRs was seen in CII_ETS_ and CIV_max_ but not in CI+II_OXPHOS _(Figure 3).

**The absolute O2 flux for each respiratory state normalized to citrate synthase (CS) activity.**
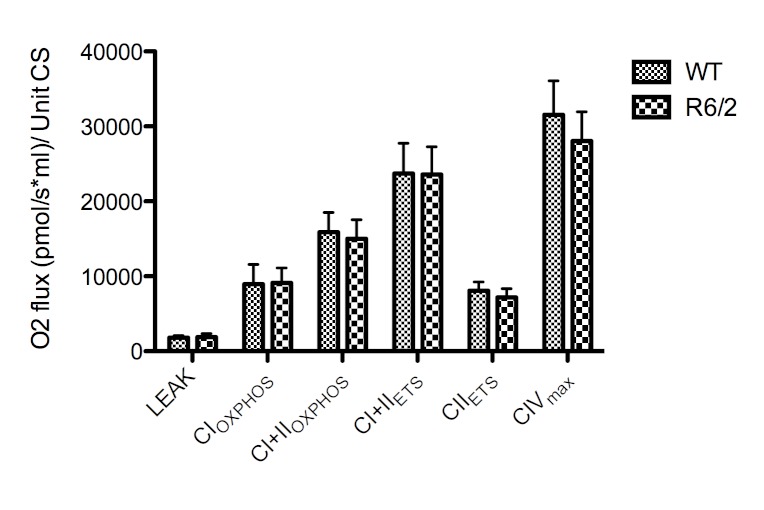
n=12 for each group. CS units are defined as µmole/ml/min. No significant difference between groups was found. Error bars depict SD.

## Discussion

This study demonstrates that the CI+II_OXPHOS_, CII_ETS_ and CIV_max_ FCRs are slightly but significantly decreased in R6/2 mice compared to age-matched wild type littermates confirming previous findings in this model [26] and in accord with several other studies demonstrating a deficiency in CII and/or CIV in other HD models 37 ^,^ 38 ^,^ 39 ^,^ 40 ^,^ 41.

The decreases in ETS function we observe are small compared to results obtained using enzyme assays. However, as our measurements include respiratory control, a meaningful comparison with results obtained using spectrophotometry is difficult. Whether the modest reduction is a cause or consequence of disease pathology is not clear, but reductions in CI activity of similar magnitudes have been reported in human cardiac failure 29.

The deficiency in CI+II_OXPHOS_ and CII_ETS_, but not in CI_OXPHOS_ or LEAK indicates a deficiency in coupled and uncoupled CII, but not CI. This difference is more pronounced in the uncoupled ETS state of CII, as electron input from CI has been inhibited and the physiological control of CII activity has been removed, which exacerbates the difference. The maximal capacity of CIV is above that of CI+CII_ETS_, with the excess capacity ensuring some degree of compensation for damage to CIV, whilst maintaining normal coupled mitochondrial respiratory function. Hence, a reduction in CIV is of limited physiological significance, unless the reduction is sufficiently severe to become the limiting factor in maximal respiration. Indeed, in some transgenic animals tested, the CIV_max_/CI+CII_ETS_ ratio was close to 1, in which case the degree of CIV inhibition could potentially limit respiration.

The mean RCR ratio was not significantly decreased in R6/2 mice compared to controls, which indicates that there is no dyscoupling of the ETS or altered mitochondrial inner membrane integrity 42. The RCR values given in this paper are estimations only, since we use LEAK (added saturating complex I substrates, but without the addition of ADP) as an expression of state 4 respiration, where the main respiratory component is proton leakage. An accurate measure of state 4 respiration would require zero ATP synthase activity, either by inhibition or a complete ATP/ADP equilibrium. As we used semi-permeabilized cells, it is likely that residual cellular ATP, ADP and cytosolic ATPases are present when CI substrates are added, which can lead to an overestimation of the RCR.

Citrate synthase is a mitochondrial marker, and assumed to reflect mitochondrial content. The CS assay is frequently used for normalisation in mitochondrial studies, but the methodological variability - as demonstrated here - can lower the statistical resolution and obscure true differences as previously reported 28 ^,^ 29, in particular when the results from two assays are combined to a ratio 43. We normalised the absolute O_2_ flux values to CS activity to investigate if this would affect our results. Indeed, the significance of the CII and CIV deficiencies were obscured by the inherent methodological variation. When all samples were pooled to get an estimate of the analytical variability of the CS assay, we found that the standard deviation was high (38.5%). The strength of HRR is the accuracy of measurements and the possibility of internal normalisation using FCRs, whereas CS is influenced by tissue heterogeneity. Furthermore, the use of CS for normalization presupposes that CS activity is not affected by the pathology of the disease studied. FCRs are completely independent of these factors. In addition, the intersample variation is much lower (10.5%) when using FCRs compared to flux/CS activity, underlining the increased sensitivity obtained by omitting an external normalisation parameter. As such, the introduction of spectrophotometry is not only unnecessary, but can obscure subtle ETS differences. We believe the explanation for the controversial nature of ETS dysfunction in the HD literature is twofold. Firstly, we believe that the insensitivity and high variability of spectrophotometric assays are partly responsible for the disagreements, which could explain the contradictions reported on the R6/2 mouse ETS function 25 ^,^ 26. Secondly, all HD disease model systems where an ETS defect has been found are based on expression of N-terminal Htt fragments, whereas full-length models exhibit no ETS defects. This leaves open the possibility that the defects are, to some degree, model effects. A number of groups have purportedly failed to reproduce ETS defects in HD models with N-terminal fragment as well as in full-length Htt, but the data mentioned are unpublished and thus difficult to evaluate 18 ^,^ 23 ^,^ 24.

A potential mechanism for the deficiency in CII and CIV function could be as a result of a reduction in cAMP responsive element binding (CREB) transcription, as mutant Htt has been shown to bind CREB-binding protein and repress transcription 44. The CYCS gene encoding CytC, which carries electrons to CIV, is CREB-dependent through cAMP response elements (CRE) 45, and could be downregulated in HD. In addition, there is a CRE element in the promoter of the CII flavoprotein subunit, which could also influence transcription 46.

In conclusion, we find that the FCRs of coupled and uncoupled CII and the CIV in the striatum of the Huntington’s disease R6/2 transgenic mouse model at 12 weeks are significantly decreased compared to wild type littermates. Furthermore, this study highlights the strengths of HRR for the evaluation of subtle differences in the ETS.

## Competing Interests

The authors have declared that no competing interests exist.
